# Emulsion Formation and Stabilization by Biomolecules: The Leading Role of Cellulose

**DOI:** 10.3390/polym11101570

**Published:** 2019-09-26

**Authors:** Carolina Costa, Bruno Medronho, Alexandra Filipe, Isabel Mira, Björn Lindman, Håkan Edlund, Magnus Norgren

**Affiliations:** 1 FSCN, Surface and Colloid Engineering, Mid Sweden University, SE-851 70 Sundsvall, Sweden; carolina.costa@miun.se (C.C.); bjorn.lindman@fkem1.lu.se (B.L.); hakan.edlund@miun.se (H.E.); 2 Faculty of Sciences and Technology (MeditBio), Ed. 8, University of Algarve, Campus de Gambelas, 8005-139 Faro, Portugal; aifilipe@ualg.pt; 3 RISE, Bioscience and Materials, SE-114 28 Stockholm, Sweden; isabel.mira@ri.se

**Keywords:** cellulose, amphiphilicity, oil–water interface, emulsion stability, adsorption

## Abstract

Emulsion stabilization by native cellulose has been mainly hampered because of its insolubility in water. Chemical modification is normally needed to obtain water-soluble cellulose derivatives. These modified celluloses have been widely used for a range of applications by the food, cosmetic, pharmaceutic, paint and construction industries. In most cases, the modified celluloses are used as rheology modifiers (thickeners) or as emulsifying agents. In the last decade, the structural features of cellulose have been revisited, with particular focus on its structural anisotropy (amphiphilicity) and the molecular interactions leading to its resistance to dissolution. The amphiphilic behavior of native cellulose is evidenced by its capacity to adsorb at the interface between oil and aqueous solvent solutions, thus being capable of stabilizing emulsions. In this overview, the fundamentals of emulsion formation and stabilization by biomolecules are briefly revisited before different aspects around the emerging role of cellulose as emulsion stabilizer are addressed in detail. Particular focus is given to systems stabilized by native cellulose, either molecularly-dissolved or not (Pickering-like effect).

## 1. Emulsion Stabilization: General Overview

### 1.1. Emulsions: Morphology and Applications

Emulsions are dispersions of one immiscible liquid into another in the form of droplets. The droplets in an emulsion are referred to as the dispersed phase, while the surrounding liquid is referred to as the continuous phase. The average diameter of the dispersed droplets can typically range from 100 nm to 100 µm. Depending on which kind of liquid forms the continuous and dispersed phases, conventional emulsions are classified as oil-in-water (o/w) or water-in-oil (w/o) [1]. “Oil” is the generic term often used for organic liquids, generally hydrocarbons, and “water” is the term used for the aqueous phase. Nevertheless, emulsions of two aqueous phases, i.e., water-in-water (w/w), can also be formed when solutions of at least two hydrophilic macromolecules that are thermodynamically incompatible in a solution are mixed, producing two immiscible aqueous phases [2]. This type of colloids has great potential in biomedical and food formulations since it can be prepared from mixtures of edible biopolymers.

Conventional o/w or w/o emulsion-based products are broadly used in our daily lives, and common examples include milk, mayonnaise, salad dressings, cosmetic and pharmaceutical lotions, creams, and waterborne paints [3,4,5,6]. Emulsion-based systems are also widely used in a range of technical applications including defoaming formulations used in the chemical and paper industries, rolling oils for metal working, explosives used in mining, asphalt, agrochemical formulations and precursor systems for dispersions and particles, just to mention as few. In the context of pharmaceutical applications, emulsions are broadly used as carrier and encapsulation systems for hydrophilic or lipophilic bioactive compounds, for which they provide protection against external stresses and enable controlled release [3,7]. The same is true for beverage applications within the food industry, where emulsions are being considered suitable carriers for the delivery of non-polar bioactive ingredients with health benefits [8,9,10].

In addition to simple o/w, w/o or w/w emulsions, more complex morphologies also exist which offer many potential improvements in terms of functional performance and are of great interest for drug/active delivery applications. Multiple emulsions are the most common examples of these so-called structured emulsions. These might be described as “emulsions of an emulsion” and could be water continuous like in the case of a (water-in-oil)-in-water (w/o/w) emulsion or oil continuous like in an (oil-in-water)-in-oil (o/w/o) emulsion. Other types of structured emulsions include multilayer emulsions, colloidosomes, solid lipid particles, microclusters, and filled hydrogel microspheres [9,10,11]. All these types of more complex morphologies are usually of less widespread use because they are more expensive and difficult to prepare. As such, they should only be considered when simpler and cheaper alternatives cannot not provide desirable functionality [9].

### 1.2. Thermodynamic Instability

Regardless of its morphology and target use, one of the main challenges faced by the emulsion formulator is to preserve the physical stability of the emulsified system. This stems from the fact that conventional emulsions are thermodynamically unstable systems and therefore tend to “break” during storage through a variety of instability mechanisms.

**Figure 1:**
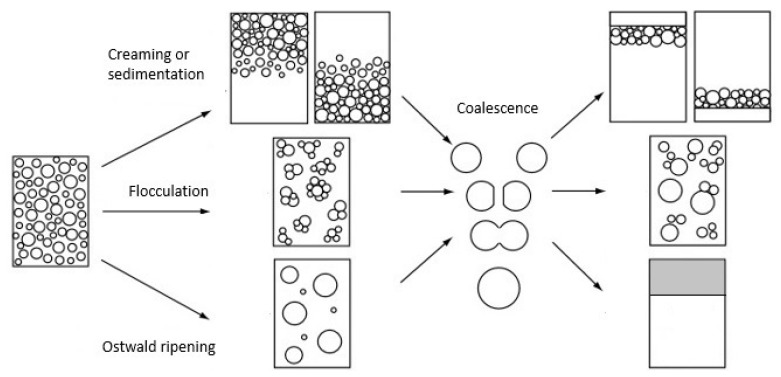
Breakdown mechanisms of an emulsion. Note that different processes may occur simultaneously. Adapted with permission from [13]. Copyright John Wiley and Sons.

The mechanisms that can lead to emulsion breakdown over time can be generically divided into: Gravitational separation, droplet aggregation (or flocculation), Ostwald ripening, and droplet coalescence (Figure 1) [12,13]. Gravitational separation arises from the difference in density between the dispersed and continuous phases, and it is referred to as “creaming” when droplets float up to the top or “sedimentation” when they sink down to the bottom of the vessel. Droplet aggregation (or flocculation) occurs when droplets attract each other, forming a loosely clumped mass of particles (“flocs”). Lastly, Ostwald ripening is caused by the difference in pressure inside large and small droplets, which leads to a mass diffusion from the smaller to the larger droplets. This phenomenon proceeds slower when the size distribution of the drops becomes narrower or when the dispersed phase is very insoluble in the continuous phase [12]. All these instability mechanisms can then lead to droplet coalescence, which is the irreversible process of two droplets merging by the disruption of the stabilizing layer forming a larger one, eventually leading to the formation of separate oil and water phases.

### 1.3. The Role of Emulsifiers and Surfactants

**Figure 2:**
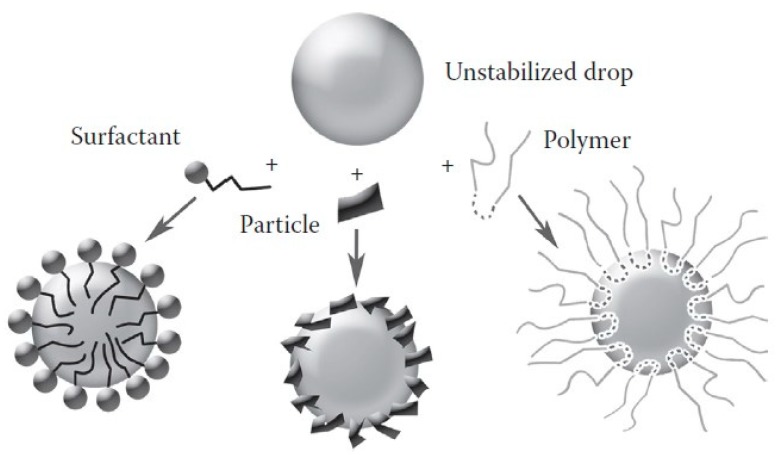
Interfacial stabilizing layers formed by surfactants, particles and polymers. Differences in scaling are not considered. Adapted with permission from [15]. Copyright CRC Press, Taylor and Francis Group.

An energy barrier needs to be created at the oil–water interface in order to prevent droplet coalescence (droplet fusion) and to attain reasonable stability, thus conferring a suitable life-time for the final emulsified product [13]. This barrier can be created through the adsorption of a stabilizing layer of amphiphilic and surface active surfactants, polymers or solid particles at the oil–water interface (Figure 2). In the context of emulsions, surfactants and emulsifiers are considered as two different types of molecules with distinct structures and physicochemical properties, and so they contribute in different ways for the emulsion stabilization. Generally, the term emulsifier is used to refer to high molecular weight polymers and colloidal particles that are capable of adsorbing at the oil–water interface and protecting emulsion droplets from aggregation or coalescence. These should ideally also rapidly adsorb at the oil–water interface and significantly reduce the interfacial tension in order to facilitate drop brake-up [7,14]. On the other hand, the term surfactant is usually used to refer to low molecular weight molecules because it is considered the most efficient in assisting emulsion formation. Due to their fast diffusion, surfactants often display a fast adsorption at newly formed interfaces. Nevertheless, for simplicity, we term all these systems as emulsifier agents in this review. Often, the combination of emulsifying agents with other stabilizing ingredients, such as rheology modifiers, may be required in order to improve the kinetic stability of the system. In this case, these thickening or gelling agents act via viscosity enhancement or gel formation of the continuous phase, thus restricting the droplets movement.

Once an emulsion is formed, it is important to keep it stable throughout the expected shelf-life of the target product. Regardless of the specific emulsion instability mechanism that the formulator is trying to counteract (gravitational separation, droplet aggregation, Ostwald ripening or droplet coalescence), it is true that the choice of emulsifier plays a very central role in attaining the target emulsion stability profile.

Sometimes, a combination of different emulsifiers is required to create a robust stabilizing layer, and many examples have been suggested including protein–polysaccharide, surfactant–protein and surfactant–polysaccharide mixtures [7,8]. In order to prevent droplet aggregation and eventual phase separation, the emulsifiers must generate strong electrostatic forces, steric repulsive forces, or a combination of both (electrosteric stabilization) that can overcome the attractive interactions between the oil droplets [13]. In the case of surfactants and polymers, an osmotic barrier is created by the presence of a high concentration of either counterions or polymer segments between the droplets, which drives the water diffusion to that area, keeping the droplets apart from each other. Regarding adsorbed polymers, there is also a repulsive force driven by the entropic penalty when polymer segments from two droplets start to entangle, since conformational rearrangements are very limited due to their high molecular weight [13].

Emulsions can be also stabilized by solid particles, forming so-called “Pickering emulsions.” The amphiphilicity of the particulate material used for Pickering emulsion stabilization is usually described in terms of surface wettability, which is measured by the three-phase contact angle of particles adsorbed at an oil–water interface [16]. The stabilization mechanism behind Pickering emulsions relies on the irreversible adsorption of particles at the oil–water interfaces, forming an effective mechanical barrier against coalescence [17]. Moreover, the energy of adsorption of homogeneous spherical particles, ΔE, follows the equation ΔE = πr^2^γ(1 − │cosθ^2^), where r^2^ is the square of the particle size, γ is the interfacial tension, and θ is the contact angle on the interface; therefore, the adsorption of spherical particles at liquid–liquid interfaces depends on both particle size and interfacial tension [18]. In other words, the bigger the particles and interfacial tension, the higher the energy of adsorption. A special type of particles known as “Janus particles” display some similarities to surfactants and polymers. They are amphiphilic particles composed of two or more faces with distinct physicochemical properties that can self-assemble in bulk media and readily adsorb to fluid interfaces, remarkably lowering the interfacial tension [19,20]. They can be synthesized in geometrically different shapes and chemical compositions with high uniformity and precision [19].

## 2. Emulsions Stabilized by Biomolecules

Most of the surface-active molecules currently used in the industry to stabilize emulsions are synthetic surfactants (e.g., sorbitan esters and ethoxylated sorbitan esters commonly known under the trade names Spans and Tweens, respectively) or animal-based emulsifiers (e.g., gelatin, egg protein, whey protein or caseinate) [8]. Today, however, consumer demands, corporative sustainability goals and regulations are driving the market towards the use of natural ingredients over synthetic ones, as well as replacing animal-based for plant-based products. With the growing global demand for the use of sustainable and “clean-label” products, the replacement of synthetic emulsifiers by renewable ones has become extremely important. Driving this change is also the fact that, besides their innate biodegradability, biomolecules also present other advantages in comparison to ones of synthetic origin, such as low toxicity, high selectivity and specific activity at high temperature, pH and salinity [21]. For these reasons, in the past two decades, researchers in the food, pharmaceutical and cosmetic industries have been putting great effort towards identifying natural alternatives from plants or microorganisms (bacteria, fungi and yeasts) for emulsion formulations [7,8,22,23]. Among the biomolecules that are currently used as natural emulsifiers or are being investigated for their potential emulsifying properties are phospholipids, biosurfactants, biopolymers and bioparticles [8]. These natural emulsifiers are surface-active biomolecules capable of adsorbing to the oil–water interface and thereby preventing emulsion droplets from coalescing. However, these biomolecules vary considerably in their ability to form and stabilize emulsions depending on their unique chemical and structural properties. Examples of the more common natural surface-active molecules already commercialized or still under investigation due to their promising function as emulsifiers are briefly revisited below.

### 2.1. Natural Emulsifiers

#### 2.1.1. Phospholipids

Globally used phospholipid-based emulsifiers in commercial products are lecithin derivatives, which are extracted from a number of biological sources such as soybeans, eggs, milk, rapeseed, canola seed, cottonseed and sunflower. Commercial lecithins typically contain a combination of various phospholipids, and their hydrophilic head-groups are typically anionic or zwitterionic, the charge strongly dependent on pH. Oil-in-water emulsions produced with sunflower lecithins have been reported to have droplet sizes of 30–160 μm when homogenized with high-shear mixers, but sizes can be reduced to 200–400 nm when using high-pressure homogenizers [8,24]. Under neutral pH conditions, lecithin-stabilized emulsions tend to display a good stability against aggregation due to a strong electrostatic repulsion of the negative charged interface [25]. However, the stability of such emulsions is highly sensitive to changes in environmental conditions, such as pH and ionic strength, due to the screening of the charges present on their surfaces [8]. The physical and chemical stability of lecithin-coated lipid droplets can be improved by coating them with oppositely charged biopolymers to form multilayer emulsions, and the same approach can be used to alter their potential gastrointestinal fate [8]. Certain types of phospholipids may also be effective at retarding the oxidation of emulsified lipids because of their natural free radical scavenging capacity [8]. The functionality of lecithins as an emulsifier can also be improved by their combination with co-solvents and other emulsifiers or by thermal, chemical or enzymatic modifications [8,26,27]. One example of the later approach are lysolecithins, which are produced from lecithin hydrolysis to obtain a more polar surfactant. Lysolecithins have been used in the food, pharmaceutical and supplement industries to produce nutritional emulsions. These emulsions are typically designed to contain mixtures of minerals, vitamins, proteins, and lipids to be taken orally or intravenously [8].

#### 2.1.2. Glycosides

Among the most commonly used natural surfactants are the saponins, which are glycosides extracted from the bark of the tree *Quillaja saponaria*. Q-Naturale^®^ is a commercial form of *Quillaja saponin* with applications in the food industry. Studies have reported that these biosurfactants form fine o/w emulsions with droplet sizes lower than 200 nm that are stable for aggregation over a wide range of pH, ionic strength, and temperatures [8]. Saponins have also been shown to protect lipid droplets from aggregation when the oil phase crystallizes, which is a particularly useful feature for the formulation of solid lipid nanoparticles or nanostructured lipid carriers [8]. Part of their ability to form stable o/w emulsions has been attributed to the formation of strong elastic interfaces that inhibit droplet deformation and coalescence [8]. Moreover, the rheological properties of the interfacial surfactant-layers are dependent on the nature of the oil phase; interfacial elasticity increases with hydrophobicity. It has been reported that vitamin-enriched nanoemulsions stabilized by *Quillaja saponins* display slower lipid oxidation rate compared to emulsions stabilized by synthetic surfactants [8].

#### 2.1.3. Glycolipids

Rhamnolipids (RLs) are the best-known biosurfactants from microorganisms. These are originally extracted from the pathogenic gram-negative bacterium *Pseudomonas aeruginosa*. Due to the outstanding surfactant ability of RLs, efforts have been made to find non-pathogenic sources to extend their application to foods, cosmetics and pharmaceutical industries. Most commercial applications of RLs are for household cleaning purposes and oil remediation strategies. A USA-based company commercializes an RL-based product called NatSurFact^®^ that can be used in a range of application areas including personal care, multi-purpose cleaning, medicine, agriculture, wastewater treatment, oil recovery and bioremediation [28]. AGAE Technologies commercializes concentrated RLs (90% and 95%) for potential use in pharmaceuticals, cosmetics, oil tank cleaning, laboratory reference standards, personal care products, home cleaning products, enhanced oil recovery and environmental remediation [29].

Another much studied family of surfactants produced by microorganisms are the sophorolipids (SLs). SLs are glycolipid biosurfactants composed of a sophorose (hydrophilic moiety) and a hydroxylated fatty acid (C_16_ or C_18_), the latter of which is the hydrophobic part. They are of interest for the food and medicine industries since they are produced from several non-pathogenic yeast genera, such as *Starmerella*, *Candida* and *Pseudohyphozyma* [30]. SLs (with mixed lactonic, acid and esterified forms) have been shown to be able to emulsify Arabian light crude oil and various pure hydrocarbon oils with a similar effectiveness to Triton X-100, a commercial nonionic surfactant [31]. Remarkably, SLs already have a place in the market for cleaning applications. REWOFERM^®^ SL is an SL-based product produced by the German-based company Evonik for use in detergents and home care cleaning products; it has received the EPAWA Innovation Award in 2016 for “the outstanding cleaning properties and the excellent ecological profile” [32]. A usual barrier for the scale-up of biosurfactants produced by microorganisms is their low yields, resulting in high production costs. Evonik was able to reduce the production costs of SLs by using genetically modified yeasts, and that methodology was granted as a US Patent in 2015 [33].

#### 2.1.4. Proteins

Amphiphilic proteins are biopolymers that have the ability to adsorb at oil–water interfaces and act as emulsifying agents. The relative balance of polar and non-polar groups exposed on their surfaces governs the surface activity of proteins and, consequently, their tendency to either adsorb to the oil–water interface, solubilize, or self-aggregate [34]. Among others, caseins, whey proteins, gelatins, egg proteins and pea proteins are commonly used in the food industry [7,8]. As discussed, small surfactants are more effective at reducing interfacial tension than proteins. However, proteins can lead to more stable emulsions due to protein–protein intermolecular interactions, which can result in highly viscoelastic films that form around the emulsion droplets [35]. Their stabilization efficiency can be improved through physical, enzymatic and genetic modifications, which allow them to expose more hydrophobic groups towards the oil phase [7,35]. Adsorbed proteins usually form interfacial films which are relatively thin (<10 nm) and electrically charged, and so the main mechanism preventing droplet flocculation is the electrostatic repulsion [8,35]. Hence, protein-coated droplets tend to flocculate at high salt levels and pH values close to their isoelectric points [8,22]. Proteins with high molecular weights and extended structures usually generate a stronger steric repulsion and are therefore better at preventing aggregation [8]. Globular proteins tend to form thicker and viscoelastic gel-like interfaces due to intermolecular cross-linking. However, they may unfold above their thermal denaturation temperature, exposing more hydrophobic and sulfhydryl groups. This, in turn, can lead to droplet aggregation due to an increased surface hydrophobicity through hydrophobic forces and the formation of covalent disulfide bonds [8,22].

#### 2.1.5. Polysaccharides

Polysaccharides are abundant biopolymers and widespread in food colloids. Because they are large molecules, their adsorption is a much slower process than for smaller surface-active agents. However, given the proper conditions that make it possible to get them all at the interface, they may provide superior stability against aggregation due to a strong steric repulsion with mainly entropic contributions [1]. The most widely used polysaccharide emulsifiers that display interfacial activity are gum arabic, pectins, galactomannans, and modified starches and celluloses [7,8,36,37,38,39,40,41]. Their interfacial activity may arise in some cases from an appropriate balance between hydrophilic and hydrophobic groups, as for modified starches and celluloses, or from the presence of glycoproteins covalently linked, which is the case of gum arabic and pectins [7]. Other polysaccharides that display little or no interfacial activity may still stabilize emulsions through structuring, thickening and gelling of the continuous aqueous phase, as in the cases of xanthan gum, alginates, carrageenans, hyaluronan and chitosan [7]. One of the major advantages of polysaccharides over other types of natural emulsifiers is their high stability against environmental stresses, since their stabilization mechanism is primarily driven by a strong steric repulsion arising from thick stabilizing layers [8,36]. On the other hand, polysaccharides usually need high polymer-to-oil weight ratio in order to produce submicron-sized droplets [37].

#### 2.1.6. Natural Colloidal Particles

The most widely used natural colloidal particles are derived from biopolymers such as cellulose, chitin and chitosan, starch and modified starches, lignin and proteins [42,43,44,45,46]. Colloidal particles can impart an outstanding stability against droplet flocculation and coalescence, and they may inhibit lipid oxidation due to the thick interfacial layers that they form [8,36]. However, bioparticles may vary widely in shape, size, aspect ratio and morphology, and some of them are made from aggregated, self-assembled or cross-linked polymers (e.g., casein-micelles, heat treated whey proteins, microgels, and partially gelatinized starch granules) [14,42,47]. This implies that their mechanistic behavior substantially deviates from that of both the solid sphere and the flexible polymer [14]. Nevertheless, particles with an irregular shape and higher aspect ratios have been found to have a greater ability in stabilizing emulsions and foams and at lower concentrations compared to particles of spherical shape [42].

## 3. The Emerging Role of Cellulose as an Emulsion Stabilizer

The exponential development of new products, including ones where the natural surface-active molecules are particularly requested due to their biocompatibility and low toxicity (e.g., food, beverages products, medicines and cosmetics), makes it necessary to continuously evaluate the efficacy of new biomolecules as putative emulsion stabilizing agents. However, the replacement of synthetic emulsifiers for natural ones can be a challenge on an industrial scale due to several factors. The source must be economically viable in terms of abundance and geographical access. It is also important that the molecules can be readily obtained in the necessary amounts without high costs. Moreover, the extraction method used to obtain a purified compound has to be cost-effective and possible to scale-up. Genetic engineering can contribute to the development of recombinant strains that are able to overexpress the desirable molecule, lowering production costs. However, the majority of the consumers are still resistant to buying products produced by genetic modified organisms (GMO). As a result, biomolecules produced from plants usually stand out in comparison to the ones isolated from microorganisms. In this context, cellulose appears as one of the key sources for sustainable materials on an industrial scale because it is the most abundant, biodegradable and renewable biopolymer on the planet [48]. It can be sourced from plants, biomass, or bacteria, and it relies on fairly simple, scalable, and efficient isolation techniques [49,50,51]. As an amphiphilic polymer, cellulose can theoretically adsorb at the oil–water interface, preventing the coalescence of the oil droplets because it is a putatively good stabilizer of emulsions. In this second part of the review, we evaluate the role and use of native cellulose as an emulsion stabilizer, either molecularly dissolved or as particles. In what follows, the physicochemical characteristics of cellulose and the importance of its amphiphilicity are revisited, and an extensive state-of-the-art of the applicability of cellulose as an emulsifier is presented.

### 3.1. Physicochemical Characteristics of Cellulose

Biopolymers are often polydisperse in molecular weight, and their chemical functionality depends on the natural source and pretreatments of the raw materials. Cellulose is a linear homopolymer of glucose residues linked by β-(1-4) glycosidic bonds. The chain length of cellulose expressed in the number of glucopyranose units (degree of polymerization—DP) in wood pulp is typically between 300 and 1700; for cotton and other plant fibers, DP values range between 800 and 10,000 [52]. In their native form, cellulose chains form parallel bundles that build up the microfibrils (containing both crystalline and amorphous domains), and these then aggregate to form cellulose fibers. Mechanical or acidic treatments are usually applied to obtain crystalline and fibrillated forms of cellulose in the submicron range like micro- or nanocrystalline cellulose (MCC/NCC) and micro- or nanofibrillated cellulose (MFC/NFC) [44,53,54,55]. ^1^H NMR spectroscopy has shown that the β-linked glucopyranose units adopt ^4^C_1_ chain conformation, which is the conformation with the lowest free energy of the molecule. Consequently, the three hydroxyl groups are located on the equatorial positions of the rings, and the hydrogen atoms of C–H bonds are located on the axial positions [56]. Therefore, cellulose molecules have an intrinsic structural anisotropy with regions of markedly different polarity, since the equatorial direction of the ring has a hydrophilic character, while the axial direction is hydrophobic [57].

Until quite recently, the consensual view of cellulose insolubility in water among the leaders in the field has been based on its ability to form a strong intermolecular hydrogen-bonding network. However, this vision contrasts with the dissolution behavior of some cellulose derivatives, like methylcellulose (MC) or hydroxyethylcellulose (HEC) and some other non-ionic polysaccharides such as dextran, all of them having good hydrogen bonding capabilities. Another clear example of this contradiction is glucose, which would show a strong tendency to self-associate and phase separate if intermolecular hydrogen bonding was the main driving force for cellulose insolubility; however, that is not the case. The behavior of complex systems always needs to be considered in light of the balance between different interactions. A more recent perspective has highlighted the amphiphilic nature of cellulose based on its structural anisotropy, and experimental research has been conducted to focus on the role of hydrophobic interactions in cellulose dissolution and regeneration [58,59,60,61,62,63,64,65,66,67]. Due to its amphiphilicity, cellulose has already been explored as an eco-friendly emulsifying agent, either in the form of micro- and nanoparticles (MCC, NCC, MFC, NFC) or through a dissolution-regeneration process of native cellulose [68,69].

### 3.2. Cellulose as an Emulsifier

Several researchers have demonstrated the ability of cellulose particles to self-assemble at oil–water interfaces and stabilize o/w emulsions without the aid of surfactants [39,70,71,72]. It is believed that the amphiphilic character of cellulose resides in its crystalline organization at the elementary brick level, and cellulose nanocrystals thus have both hydrophilic and hydrophobic edges that are preferentially wetted by water and oil phases, respectively [71]. The mechanism by which solid particles self-assemble at oil–water interfaces and form a protective layer around the emulsion droplets is known as the Pickering mechanism, which was introduced by Pickering in 1907 [73]. Both o/w and w/o emulsions can be formed depending on particle wettability and whether the particles are predominantly hydrophilic or hydrophobic [17]. If it has a high enough concentration of particles in the system, the interface tends to bend towards the poorer wetting liquid, and this becomes the dispersed phase. The wettability properties of cellulose particles may be tuned by surface hydrophobization, since the surface of cellulose contains many reactive hydroxyl groups, and w/o emulsions can be formed [74,75,76]. Most of the particles from biological origins, such as cellulose, chitosan, or starch, show an irregular shape and are polydisperse in size and morphology, which makes them difficult to characterize. Nevertheless, this structural anisotropy may be very beneficial for emulsion stability. Madivala et al. found that particles with high aspect ratios are capable of stabilizing biphasic systems at lower concentrations compared to those with spherical shapes [77]. Particles with such a well-defined shape are usually derived from inorganic materials, like silica. Silica particles have been extensively studied because of their availability in different sizes with narrow distributions and chemical surface tunability. However, their lack of biocompatibility and biodegradability restricts their use in food and pharmaceutical applications [78]. For this reason, the study and characterization of materials from biological origins have gained increasing attention, and many efforts have been made in the food and pharmaceutical industries in order to develop new food-grade particles [42,79].

Early studies performed by Oza et al. not only demonstrated the ability of MCC dispersions to stabilize conventional o/w emulsions, they also demonstrated multiple emulsions systems of the w/o/w type with the aid of a hydrophobic surfactant for the stabilization of the internal w/o interface [80,81]. Moreover, Oza et al. reported that MCC particles form a network around the emulsified oil droplets that provides a mechanical barrier against coalescence, and, beyond that, the non-adsorbed particles act as thickener agents in the continuous aqueous phase. MCC particles have also been used in w/o Pickering emulsions to reduce lipid oxidation, one of the major concerns among food manufacturers due to its negative effects on food quality [79].

More recently, cellulose nanoparticles have also shown their effectiveness at stabilizing emulsions [68]. It has been found that less than 0.1% (w/w) of sulfated cellulose nanocrystals from cotton are sufficient to form stable o/w emulsions known as HIPEs (high internal phase emulsions) that contain volume fractions of oil as high as 0.9 [72]. However, NCC produced with sulfuric acid has been shown to bear sulfate groups with a surface charge density of 0.123 e/nm^2^, which was found to not be beneficial for the stability of emulsions. The anionic charges on the surface of the nanocrystals repel the neighboring adsorbed particles, which may induce a negative effect on the packing of the particles at the oil–water interface. To prevent electrostatic repulsions, the charges may undergo desulfation or may be screened by the addition of a certain amount of salt [71,72]. Kalashnikova et al. reported that a surface charge density lower than ca. 0.03 e/nm^2^ is ideal for the effectiveness of NCC as an emulsifier agent [71]. The authors concluded that the electrostatic interactions play a major role in the control of the exposure of the hydrophobic edge planes to the oil interface.

Another interesting work reported on ammonium persulfate hydrolysis for the preparation of NCC modified with carboxylic acid groups [82]. The resultant o/w emulsions showed good stability against creaming, which was observed to be enhanced by the increase in NCC concentration. Furthermore, parameters such as temperature, ionic strength and pH were varied to study emulsion stability against external conditions. Creaming increased at a low pH or a high salt concentration due to the electrostatic screening effect. On the other hand, the stability to creaming was improved by an increase in temperature from 20 to 70 °C. It was suggested that NCC particles were irreversibly adsorbed at the interface with a temperature increase, forming a 2D-network around the droplet surface.

Double emulsions consisting of o/w/o using a combination of native and hydrophobized NCC and NFC have also been reported with average diameters ranging from 43 to 76 μm [83]. The authors mention that the large size of the outer droplets makes the encapsulation of hundreds to thousands of inner oil droplets of ∼3 µm possible. Moreover, the average size of the droplets can be tailored by selecting specific combinations of cellulose nanoparticles. It should be noted that double emulsions stabilized by cellulose nanoparticles have shown remarkable stability (over a month).

Winuprasith et al. prepared stable emulsions using MFC from mangosteen rinds [84]. Microscopic observations revealed adsorbed MFC particles at the oil–water interface regardless of their concentration. Additionally, the excess of non-adsorbing particles, at higher concentrations, was observed to induce a 3D-network formation in the continuous phase, which was found to be important for stability improvement. All emulsions were found to be stable against coalescence for a period of 80 days, but stability against creaming decreased for low MFC concentrations (<0.5%). Interestingly, the droplet size was observed to increase with MFC concentration, while the opposite effect was observed before for NCC. The authors explained this behavior by highlighting the structural differences between MFC and other particles of lower aspect ratios. During emulsification, the weakly bonded network of the particles that had a low aspect ratio fell apart easily, and individual elements started to flow, resulting in a decrease in viscosity. On the other hand, the more entangled network of MFC gradually broke, and at a sufficiently high shear rate, the suspension flowed as individual flocs and its viscosity remained high, thus reducing the efficiency of the emulsification process [85,86].

**Figure 3:**
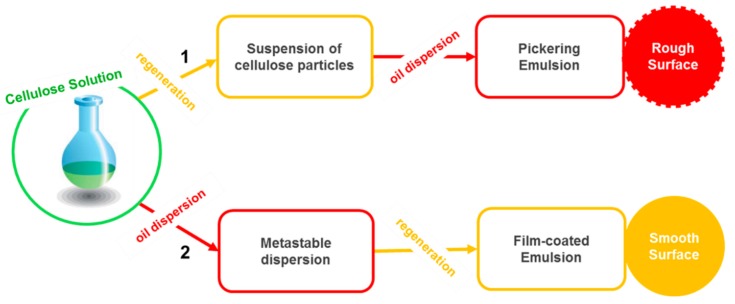
Two established approaches of preparing emulsions stabilized by cellulose. Adapted from [91].

Apart from the use of cellulose (nano)particles (crystals and fibrils), another interesting and promising approach is based on the use of native cellulose processed via dissolution and regeneration steps to stabilize emulsions. The literature in this reasonably new area is still scarce, but the available reports suggest some important benefits over the use of cellulose (nano)particles regarding preparation methods and yields. For instance, one important drawback is that during NCC preparation, the hydrolysis of the amorphous domains results in a significant loss of the cellulose biomass, and the yield of NCC is usually lower than 30% for already optimized protocols [87]. Furthermore, the mechanical methods used to prepare micro-/nanofibrillated cellulose require extremely high energy inputs (ca. 25 000 kWh per ton in the production of MFC) due to the multiple passes through the homogenizers, thus making the overall process economically nonviable. Additionally, the extensive clogging of the homogenizer has been found to be a chronic problem limiting the scaling up of NFC or MFC production [53]. On the other hand, regenerated cellulose can easily be prepared by a dissolution–regeneration process, yielding more than 80% of the original cellulose biomass [88]. The regeneration step in cellulose processing occurs when a coagulant (“anti-solvent”) gets in contact with a cellulose dope, leading to solvent exchange and the aggregation of the cellulose chains. The organization of the molecules in the regenerated aggregates and the properties of the regenerated materials (fibers, films, foams, particles, etc.) are strongly influenced by the dissolution state of cellulose, i.e., if dissolved cellulose is molecularly dispersed (or close to it) or in cellulose aggregates (crystallites) [66,89]. Rein et al. were the first to report on the use of dissolved cellulose as the starting point for the production of emulsions [69]. They used two different approaches for creating emulsions, starting from a cellulose solution in the ionic liquid 1-ethyl-3-methylimidazolium acetate (EMIMAc), namely: (1) The coagulation of cellulose with water prior to the dispersion of the oil; and (2) the dispersion of the oil into the cellulose solution, followed by the coagulation of cellulose at the interface with water (the in-situ regeneration of cellulose) (Figure 3) contains a schematization of the steps involved in the two methods). Shortly afterwards, Jia et al. reported on emulsions prepared via the former approach using an 85 wt% phosphoric acid solution in water as a solvent for cellulose [88,90]. The emulsions produced by both methods were reported to display very good stability against droplet coalescence.

**Figure 4:**
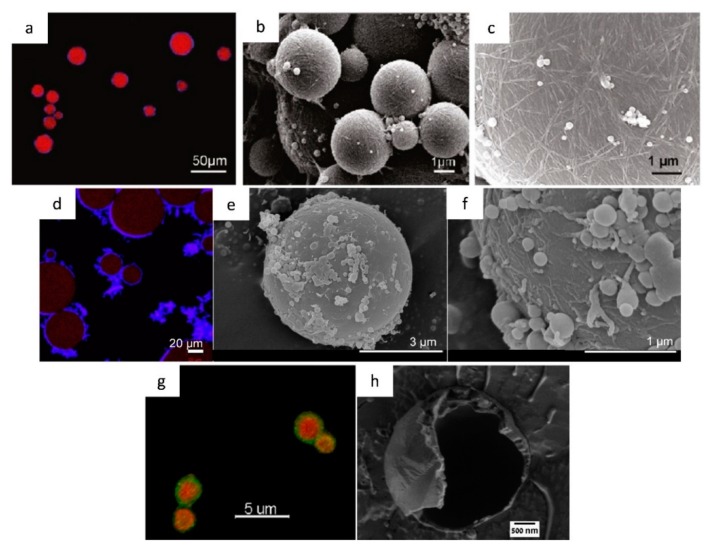
(**a**) Confocal laser scanning micrograph of hexadecane droplets stabilized by bacterial cellulose nanocrystals (BCN) with double staining (oil stained with BODIPY564/570 and BCN stained with calcofluor white). (**b**,**c**) Scanning electron micrographs of styrene-polymerized Pickering emulsion stabilized by BCN. (**d**) Confocal laser scanning micrograph of citrus oil droplets stabilized by regenerated cellulose with double straining (oil stained with Nile red and cellulose shell stained with calcofluor white). (**e**,**f**) Scanning electron micrographs of styrene-polymerized emulsion stabilized by cellulose regenerated from an 85 wt% phosphoric acid solution. (**g**) Fluorescence microscope image of double stained cellulose-coated emulsion droplets of n-hexadecane/toluene (1/1) (oil mixture stained with Nile red and cellulose shell stained with calcofluor white). (**h**) Cryo-SEM image of the fractured surface of an empty cellulose-shell of an emulsion prepared from a molecular solution of cellulose in 1-ethyl-3-methylimidazolium acetate (EMIMAc). Adapted with permission from [70] and [93]. Copyright 2011 and 2018 American Chemical Society.

In the first approach, particle-stabilized emulsions are produced by regenerating cellulose from a solution and then dispersing the oil in the resulting suspension of cellulose particles. Jia et al. suggested that the mechanism behind droplet stabilization in emulsions prepared in this way is similar to the one for emulsions stabilized by cellulose nanoparticles as described above, i.e., a combination of particle adsorption (Pickering stabilization) and network formation in the continuous phase [44,68,82,83,90,92]. The occurrence of these two stabilization mechanisms, when stabilizing different emulsion systems, is displayed in Figure 4a–c for bacterial cellulose nanocrystals (BCN) and in Figure 4d–f for regenerated cellulose.

On the other hand, in the second approach, the mechanism of drop stabilization appears to be different. In this case, the drops seem to be stabilized by a “cellulose continuous smooth shell” that forms as the regeneration of cellulose takes place on the surface of the oil droplets, as can be seen in Figure 4g,h [69,93]. Apart from differences in the features of the resulting droplets, there is another fundamental difference between the two described emulsifying mechanisms. This difference has to do with the existence of dissolved cellulose during the initial stage of emulsification in the second approach. This aspect, not addressed in detail in any of the previous studies, was the focus of one of our recent works [91]. The interfacial activity of cellulose (dissolved in an 85 wt% phosphoric acid solution) prior to in-situ regeneration and its influence on the properties of o/w emulsions produced before and after cellulose regeneration was investigated. It was found that dissolved cellulose was able to significantly decrease the interfacial tension between the oil and water phases in a comparable magnitude to that displayed by non-ionic cellulose derivatives [94]. Additionally, recent molecular dynamic simulations indicated that molecularly dispersed cellulose gradually assembles at the oil–water interface, eventually surrounding the oil droplet [95]. However, the properties of molecularly dissolved cellulose in creating and stabilizing emulsions are clearly much less explored due to its well-known dissolution limitations.

In its molecularly dissolved state, cellulose has proven to facilitate the dispersion of the in the aqueous-phase, but emulsions are not stable, showing coalescence after a few hours [91]. This instability has been ascribed to the polar character of cellulose molecules in extreme acidic conditions (protonation). However, emulsions that are highly stable against droplet-coalescence are formed when amphiphilicity changing cellulose by a slight increase in pH when adding water in a second step of emulsification. Finally, the ageing of cellulose solutions before emulsification has been found to favor the creation of smaller droplets due to a reduction in the molecular weight of cellulose.

The formulation and relevant characteristics of the emulsions stabilized by regenerated cellulose are summarized in the Table 1. In all cases, emulsions have been found to be very stable against coalescence due to the irreversible adsorption of the cellulose onto the droplet surface. This has been supported by different microscopy techniques [88,90,96].

In the cases where cellulose concentrations have been below 0.8% (w/w) and high shear forces have been applied for the mixing and homogenization of the emulsions, creaming has been typically observed after a couple of months (Table 1). The creaming rate has been found to decrease when increasing the cellulose concentration. Nevertheless, the concentrations required to delay creaming are lower than those of common food-grade polysaccharides, such as modified starch (15–25 g/kg) [79]. When sonication has been applied instead of mechanical shear homogenizers, no creaming effects have been observed within one year, regardless the concentration of cellulose used. The formation of fine droplets by ultrasound has contributed to slowing down the creaming rate, since smaller droplets experience less gravity effects. Droplet structural features always depend on the device used for emulsification, operation conditions (i.e., input energy, time and temperature), and formulation. Smaller and monodisperse droplets tend to have superior stability.

Interestingly, a 3D network formation was also found to play a key role in stabilization. Jia et al. and Shen et al. showed that the resulting emulsions are shear-thinning with typical gel-like characteristics, which contributes to the decrease in droplet mobility [90,96]. The authors concluded that emulsion stabilization by regenerated cellulose is a combination of particle adsorption (Pickering stabilization) and network formation in the continuous phase that is provided by non-adsorbed cellulose. In addition, the formed emulsions have been found to be remarkably stable against changes in external conditions such as pH, ionic strength, and temperature; this makes them good candidates for target delivering in complex conditions, and it presents an important advantage over negatively charged NCC [96].

Another work reported on the use of sodium-caseinate (SC) as the primary emulsifier of o/w emulsions and regenerated cellulose as a co-stabilizer [97]. SC is a protein extracted from milk, and it is commonly used as an emulsifier in the food industry [98,99,100]. The authors found that the concentration of regenerated cellulose affected the protein adsorption at the oil–water interface, which then resulted in a significant drop in size of the emulsions and an enhancement of their stability. The amount of adsorbed proteins was increased from 8.25 to 9.71 mg/m^2^, while regenerated cellulose was increased from 0% to 1.5%. At 1.5% (w/w), a strong network was formed, which contributed to a stability enhancement in creaming and droplet flocculation. This interesting observation should be further explored because proteins are an important class of food emulsifiers, and regenerated cellulose may therefore be used to improve the physical and functional interfacial properties of protein layers.

## 4. Conclusions

It is clear that formulations containing emulsions occupy a prominent place in different areas. Their impact on our daily life is remarkable, and it is therefore not surprising that natural biomolecules have been emerging as important players to partially or completely replace the available non-sustainable options. The list is of formulations is vast, but the future leading role of cellulose as an effective stabilizing agent is unquestionable and opens a new era for the development of sustainable, biocompatible, and value-added functional materials. Surfactant-free emulsions, containing relatively small droplets and low cellulose concentrations, have recently been developed using all forms of cellulose (crystalline, fibrillated, and regenerated) that provide a strong support for the vision of cellulose as an amphiphilic molecule. Structural differences and advantages/disadvantages between different cellulose forms have been presented in this review. Cellulose particles—crystals, fibrils, or regenerated—self-assemble at the oil–water interface to form a fiber-like coating around the oil droplets that acts as a powerful mechanical barrier against coalescence and lipid oxidation. A second mechanism of stabilization is provided by the non-adsorbed particles in the continuous phase, and these particles form a 3D interconnected network that entraps oil droplets and further reduces their movement. The colloidal assembly of cellulose particles when liquid interfaces of notably different polarities are present might serve as a template for the synthesis of new functional microcapsules. Regenerated cellulose presents important advantages over nanocrystalline and fibrillated particles (NCC, MFC and NFC) regarding preparation methods and yields. Moreover, its hydrophilic–lipophilic balance can be tuned by playing with solvent quality and regeneration coagulant. As such, it deserves more attention in the future. Additionally, it can be a potential candidate for target delivery in complex conditions due to its exceptional stability against environmental stresses (pH, ionic strength and temperature). Overall, our brief treatise reinforces current demands in replacing synthetic emulsion stabilizers with natural ones, highlighting the leading role of cellulose in this expected transition. Learning how to use biopolymers with minimal chemical modification is of great relevance for the continuous development of sustainably engineered bio-based materials.

## Figures and Tables

**Table 1:** Brief summary of the formulation and characteristics of emulsions stabilized by regenerated cellulose.

Cellulose Source/Concentration	Co-stabilizer/Concentration	Solvent	Oil Type/Concentration	Homogenization Method	Emulsion Characteristics				Ref.
					Type	Droplet Size	Rheological Properties	Stability	
MCC powder (no specification)	--	EMIMAC	n-Eicosane 5% w/w	Sonication	Oil-in-water Water-in-oil	200 nm–20 µm	--	- No reversible flocculation or irreversible coalescence is observed within 1 year for o/w emulsions. - w/o emulsions are less stable, and phase separation is observed after several months.	[69]
MCC powder 0.7%–4% w/w *	--	EMIMAC	Paraffin	High-shear (Ultra turrax) followed by Sonication	Oil-in-water	20 µm ^(a)^	--	--	[101]
MCC powder 0%–1.06% w/v **	--	85% w/w Phosphoric acid aq. solution	Dodecane 10% v/v	High-shear (Ultra turrax)	Oil-in-water	20 µm (concs > 0.11%)	--	- No coalescence (concs. > 0.11%). - No creaming (concs. > 0.84%).	[88]
MCC powder 0.07%–1.10% w/v **	--	85% w/w Phosphoric acid aq. solution	Dodecane 25% v/v	High-shear (Ultra turrax)	Oil-in-water	20–40 µm	Gel-like; Viscosity increase during storage	- No coalescence. - No creaming within 3 months (concs. > 0.83%).	[90]
MCC powder 3–8 g/kg ** (0.3%–0.8% w/w)	--	85% w/w Phosphoric acid aq. solution	Citrus oil 5 to 20mL/L (0.5%–2.0% v/v)	1. Sonication 2. High-shear (Ultra turrax)	Oil-in-water	1–3 µm 4–9 µm	Gel-like	- No coalescence. - No creaming within 1 month.	[96]
MCC powder 0%–2.0% w/w **	Sodium caseinate 2.0% w/w *	85% w/w Phosphoric acid aq. solution	Soybean oil 30% w/w	High-shear (Ultra turrax)	Oil-in-water	14–32 µm	Gel-like; viscosity increase during storage	- No creaming within 7 days (concs. > 1.0%). - Depletion flocculation prevented for cellulose concentrations above 1.5%.	[97]
Sulfite dissolving pulp 0.1% w/w ***	--	85% w/w Phosphoric acid aq. solution	Paraffin	Sonication	Oil-in-water	2–5 µm	--	- No coalescence. - Reversible flocculation and creaming within 1 day.	[91]

* Concentration of the regenerated particles in the initial cellulose/IL solutions. ** Concentration of the regenerated particles in the total emulsion. *** Concentration of dissolved cellulose in the initial solution. ^(a)^ At the optimal conditions: 0.7%–4% w/w at 70 °C and oil/cellulose mass ratios between 1:1 and 1.5:1.
